# Training strategies and outcomes of *ab interno* trabeculectomy with the trabectome

**DOI:** 10.12688/f1000research.10236.2

**Published:** 2017-05-02

**Authors:** Katherine Fallano, Igor Bussel, Larry Kagemann, Kira L. Lathrop, Nils Loewen

**Affiliations:** 1 Department of Ophthalmology, University of Pittsburgh School of Medicine, Pittsburgh, PA, USA; 2 Food and Drug Administration, Silver Springs, MS, USA; 3 Department of Ophthalmology, New York University School of Medicine, New York, NY, USA

**Keywords:** Glaucoma, training, microincisional glaucoma surgery, ab interno trabeculectomy, trabectome, canalogram

## Abstract

Plasma-mediated *ab interno* trabeculectomy with the trabectome was first approved by the US Food and Drug Administration in 2004 for use in adult and pediatric glaucomas. Since then, increased clinical experience and updated outcome data have led to its expanded use, including a range of glaucomas and angle presentations, previously deemed to be relatively contraindicated. The main benefits are a high degree of safety, ease, and speed compared to traditional filtering surgery and tube shunts. The increasing burden of glaucoma and expanding life expectancy has resulted in demand for well-trained surgeons. In this article, we discuss the results of trabectome surgery in standard and nonstandard indications. We present training strategies of the surgical technique that include a pig eye model, and visualization exercises that can be performed before and at the conclusion of standard cataract surgery in patients who do not have glaucoma. We detail the mechanism of enhancing the conventional outflow pathway and describe methods of visualization and function testing.

## Introduction

The trabecular meshwork (TM) is the main resistance of the conventional outflow route of aqueous humor in primary and - to an even greater extent - in secondary open angle glaucoma ^1^. Several procedures and devices to bypass or ablate the TM exist. The main differences are the amount of access to angle structures measured in degrees of angle arc, method of TM removal (ablation in ab interno trabeculectomy (AIT) versus disruption), and whether an implant remains in the eye or not.

Suture or catheter trabeculotomy in gonioscopy-assisted transluminal trabeculotomy (GATT) and Trab360 (Sight Sciences, Menlo Park, CA, USA) can disrupt 360 degrees of TM through a single access site, whereas trabectome surgery (Neomedix Inc., Tustin, CA, USA) or goniotomy can achieve near 180 degrees of TM ablation or incision through a single clear corneal wound. While the use of a second site can increase ablation to 360 degrees, an 180-degree ablation provides additional flow approximately 30 degrees beyond each ablation endpoint ^2, 3^. The resulting circumferential flow can be detected experimentally and occurs even on the opposite site of the ablation ^4, 5^.

A key feature of the trabectome is a ramping “footplate that provides the key function of lifting the TM and putting it on a slight stretch, positioning the tissue for maximal discharge effect from above while protecting underlying tissue” ^6^. This device was described 15 years ago by Baerveldt and Chuck ( http://www.google.com/patents/US6979328) and US Food and Drug Administration approved on February 9, 2004, for the treatment of adult and pediatric glaucoma ( http://www.accessdata.fda.gov/cdrh_docs/pdf4/K040584.pdf). It represents the refinement of a mechanical goniectomy instrument ( http://www.google.com/patents/US6979328). While the plasma created at the tip of the electrosurgical trabectome molecularizes the TM and is the more atraumatic and drag-free TM removal technique, it does require a high frequency generator that is not necessary with the goniectome ( http://www.google.com/patents/US6979328) or the dual blade introduced a few years later ( https://patents.google.com/patent/US20150297400A1/en). This device has recently been rekindled for use in operating rooms where a high-frequency generator is not available ^7, 8^. Preclinical investigations on ab interno trabeculectomy with the trabectome and a dual-blade device demonstrated a similar decrease in intraocular pressure (IOP) in an eye perfusion model ^8^. A key differentiator among these instruments, however, is the active irrigation and aspiration system that facilitates visualization, a challenge that is well known from the pediatric goniosurgery literature ^9– 11^.

**Figure 1.**
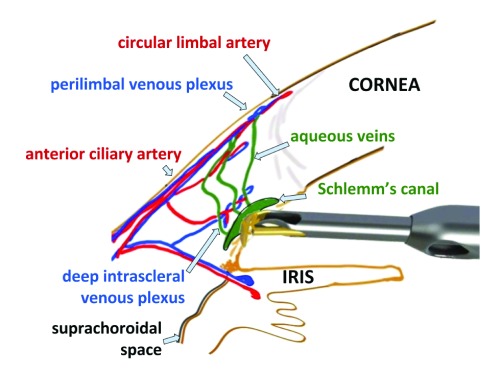
Trabectome tip inserted in Schlemm’s canal. Vulnerable structures in proximity are the iris root, the suprachoroidal space, the cornea and the deep intrascleral venous plexus.

Endoscopic excimer and YAG-laser trabeculotomy are limited to only a few circular TM ablation spots because the probe has to touch and ideally be parallel to the TM. This can be best achieved with the tip resting against the chamber angle opposite to the insertion site. TM micro-bypass implants (e.g. the iStent, Glaukos, Laguna Hill, CA, USA) are similar to such laser trabeculotomy by creating a single lumen access with an outflow enhancement over approximately 60 degrees of angle structures ^2, 3^ unless several implants are used, or a longer scaffold is inserted ^12, 13^. Compared to epibulbar drainage implants, angle surgery places unique demands on surgeons, due to the highly confined space of the angle that is approximately 200 fold smaller. Vulnerable structures are in proximity to the TM ablation ( Figure 1) and consist of the deep intrascleral venous plexus, aqueous veins, mid limbal intrascleral plexus and vessels of the iris root ^14^. Injuring those can present postoperative challenges and discourage new surgeons. It is, therefore, important to be persistent in learning the proper technique. In this article, we discuss AIT specifically with the trabectome (Neomedix, Inc.) and consider a method from other angle surgeries, a trabecular microbypass. In addition, we present a training model that has evolved from a pig eye research system and uses fluorescein or fluorescent spheres to trace or quantify outflow, and we discuss how many eyes are required to become a safe surgeon and why three to four times more eyes are needed to learn how to master this surgery. A guide to practice angle visualization and mock techniques prior to or at the conclusion of cataract surgery is presented, to help trainees who do not have access to pig eyes.

## Surgical and training methodologies

### Surgical techniques

**Figure 2.**
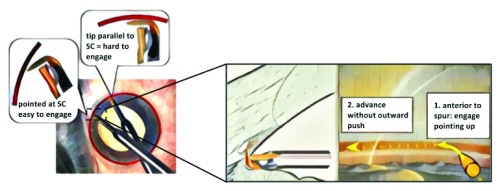
Technique to enter Schlemm’s canal. The TM is engaged toward the left and with a 45 degree upward stroke before straightening out after Schlemm’s canal is entered.

AIT is performed first for optimal angle visualization. A 1.8 mm wide iris planar clear corneal incision is fashioned approximately 2 mm anterior to the surgical limbus. No viscoelastic is used at this stage as it can contribute to carbonization during ablation. The patient’s head is rotated about 40 degrees away from the surgeon, and the microscope is tilted in the opposite direction. The ideal gonioscopic TM visualization is achieved when the angle between the microscope and the patient’s eye is about 70 to 80 degrees. The incision is gaped to induce hypotony and enable identification of Schlemm’s canal from refluxed blood. It is also possible to use Trypan blue to stain the TM if desired ( https://www.google.com/patents/US6372449). If the anterior chamber is too shallow for a full insertion, the irrigation ports of the metal sleeve allow forming the anterior chamber by resting the ports against the outer lips of the incision while the tip is already inside of the eye. The trabectome is engaged in the TM with the tip pointing 45 degrees upward just anterior to the scleral spur for a more pointed entry into the meshwork ( Figure 2); a slightly offset approach towards the left further facilitates the engagement. The trabectome is then advanced parallel with no outward push toward the wall of the canal. The handpiece is turned 180 degrees to complete the clockwise ablation. By tilting the goniolens toward the brow and then toward the cheek (or in inverse order depending on whether the right or left eye is operated on), it is feasible to visualize the superior and inferior angle structures and remove nearly 180 degrees of meshwork. After removing the trabectome, viscoelastic is injected to pressurize the eye. If proceeding with cataract surgery, the incision is enlarged with a regular keratome. A more detailed explanation of this technique can be found in Polat and Loewen ^15^.

### Training strategies and visualization of the outflow system

**Figure 3.**
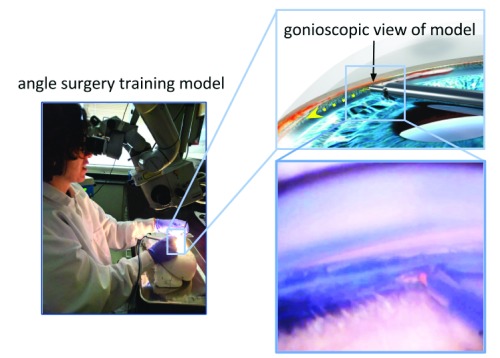
Angle surgery training model using pig eyes. The eye is mounted in a mannequin head and the angle is visualized with a surgical microscope using a goniolens (left). Gonioscopic view and tactile feedback are very similar to human eyes, except that Schlemm’s canal like segments (angular aqueous plexus) are present instead of a continuous, single lumen that is often seen in humans. Please see the following references for more information ^3, 4, 22^.

Microincisional glaucoma surgeries (MIGS) ^16, 17^ occur in a space that is approximately 200-fold smaller than what is used during implantation of epibulbar glaucoma drainage devices ^18^, making them challenging to learn. The iris root, ciliary body band, suprachoroidal space, and the deep venous plexus distal to the outer wall of Schlemm’s canal are structures that can become injured with variable sequelae ^19, 20^. Mastery of this surgery hinges on becoming proficient at visualizing the angle, identifying the correct target, avoiding trauma and maximizing the ablation length. Unfortunately, commonly used simulators or model systems with synthetic eyes in cataract surgery wet labs are not available for MIGS. Therefore, most aspiring MIG surgeons practice on glaucoma patients, even though reported complications are nearly tenfold as high as toward the end of the learning curve in ophthalmic surgery ^21^. For this reason, we developed a safe and low-cost training environment that uses pig eyes mounted into a model head ( Figure 3; ^3, 4^), which permits the tracking of progress objectively. In these eyes, outflow is traced by infusion of diluted fluorescein (0.017 mg/ml; ^3– 5^), used for intravenous applications in ophthalmic angiography or from the bottle used for tonography, although the latter source contains a preservative that may change diffusion barriers. Fluorescein has the advantage of diffusing through the TM, allowing estimation of flow speeds in non-ablated parts of the eye. A downside is that diffusion occurs over time also through intact vascular endothelium to stain the extravascular space. This is not the case within the 15 minutes of outflow tracing when fluorescent spheres of 0.5 microns are used (100-fold dilution of FluoSpheres Carboxylate-Modified Microspheres, 0.5 µm, yellow-green fluorescent (505/515), 2% solids, Thermo Fisher Scientific, Eugene, OR, USA; ^4, 22^). Fluorescent beads provide a less time sensitive, beginner-friendly method of quantifying the extent of ablation, but limits the user to a semi-quantitative assessment that does not provide flow speeds or volume estimates as fluorescein does.

### Outflow tract reconstruction with SD-OCT

**Figure 4.**
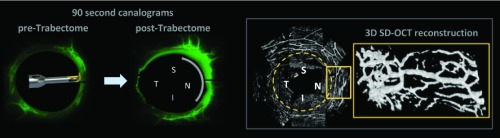
Pre and post-trabecular ablation canalogram example after trabectome surgery in our training model (left). Patency of outflow structures can be visualized in this pig eye model with spectral domain-optical coherence tomography (SD-OCT). Please see the following references for more information ^3– 5, 22^.

Aqueous spaces, but less so actual flow function, can also be reconstructed using spectral domain-optical coherence tomography (SD-OCT). Past protocols were time consuming and required manual curation and delineation ^23^. Consequently, we developed a new, automated method that uses a SD-OCT optics engine (Bioptigen, Research Triangle, Durham, NC, USA) coupled with a wide-bandwidth diode array (870-nm center wavelength, 200-nm bandwidth; model Q870; Superlum Ltd, Dublin, Ireland). If using an *ex vivo* model, the eye is placed into a holder with the eye facing up. If a patient’s eye is imaged, it is best to provide a fixation target. The head of the SD-OCT has to be steadied with a mount during the scan to prevent motion artifacts. After obtaining individual radial scan sets, each clock hour is imaged with a density of 512×512 axial scans to acquire a 2 by 3 mm area of tissue. After pre and post image processing, 3D rendered stacks can be assembled and segmented using Fiji/ImageJ (ImageJ 1.50b; http://imagej.nih.gov/ij, Wayne Rasband, National Institutes of Health, Bethesda, MD, USA) ^24^. This automated segmentation protocol creates a virtual cast of the aqueous outflow tract ( Figure 4, right).

Depending on the equipment that is available, an *ex vivo* training system might be too challenging to implement. Our team has found the following a useful technique for setting up a simulation in cataract patients: 1) positioning the patient’s head; 2) setting up the microscope; 3) gonioscopic visualization of the angle; and 3) induction of blood reflux to identify Schlemm’s canal. The headrest needs to be close enough to the stand of the main body of the microscope to accommodate the tilted view and greater distance from it. Trainees can tell their patient that they would like to take a brief look at the angle of the eye. With the surgeon seated temporally, the patient's head is tilted away by 30 degrees. The microscope is set up by centering the head of the microscope, confirming that the tilt knob is covered with a handle and then tilted toward the surgeon by 30 degrees. The microscope is lowered manually to bring the limbus into focus. Practicing gonioscopic visualization of the angle requires confirming the proper handedness of the modified Swan Jacob gonioprism, placing it on the eye and moving the microscope’s focus down toward the nasal angle. The iris root, ciliary body band, trabecular meshwork can be seen and needs to be distinguished from Schwalbe’s line and Sampaolesi line. Using a 0.12 forcep to tap lightly on the posterior lip of the primary cataract incision, induction of blood reflux identifies Schlemm’s canal. After several seconds, the goniolens is placed onto the cornea again to visualize a partially venous blood filled Schlemm’s canal.

## Outcomes from trabectome surgery

### Mechanism of outflow enhancement

By removing the primary outflow resistance (the TM) the aqueous humor can more easily pass into the collector channels and aqueous veins ( Figure 4). According to the Goldmann equation (Intraocular Pressure = [Aqueous Humor Formation/Outflow] + Episcleral Venous Pressure), this should cause the IOP to drop to the level of the episcleral veins ^25^. However, this is rare and most postoperative IOP average are around 16 mmHg ^26^. The change of diameters, collapse or patency of collector channels can be imaged using SD-OCT ( Figure 4, right; ^27^). Similar to human eyes, pig eyes have more outflow along the nasal drainage system compared with the temporal angle; nasal TM ablation is able to enhance outflow beyond physiological levels and causes fluorescein to flow circumferential through small connections between Schlemm-canal like segments that are characteristic for the angular aqueous plexus of the pig ^3– 5^. New reconstruction of the outflow tract via SD-OCT confirmed that the presence of aqueous spaces matched collectors where flow was seen. However, non-perfused vascular structures exist that might belong to the arterial or venous vascular system or reflect poorly perfused collector channels.

Using the pig eye training system mentioned above, we previously found that surgical time decreases by 1.4 minutes per eye in a linear fashion, and the ablation arc length follows an asymptotic function with a half-maximum after 5.3 eyes, while an operating room score achieves a half-maximum already after only 2.5 eyes ^22, 28^. This rapid improvement is contrasted by a slower slope of outflow in canalograms in this model, and suggests that achievement of true mastery requires about 29 eyes ^22^.

Recent discoveries indicate valve-like structures that appear to guard the collector channel orifices and collapsible aqueous veins ^29, 30^, calling into question the simplistic view that collector channel openings are round and unobstructed. Structural data indicates that flaps are kept in suspension through string-like attachments. This suggests that either these attachments should be maintained or that the flaps at the opening of collector channels need to be removed. It is not proven which procedures do this, but it is likely that a longer scaffold that displaces the TM away from the outer wall will consequentially disrupt the flap attachments and allow the flaps to remain. Electron microscopic images of the outer wall from trabectome ablation suggest that most of them are removed together with their attachments and the TM ^27^.

### Indications and patient outcomes

***Phacoemulsification combined with AIT.*** As cataract and glaucoma often co-exist in the same patient, phacoemulsification combined with trabectome surgery is a useful and cost-effective option for the treatment of both conditions ^24^. Combined phaco-trabectome surgery can result in an approximate 18% reduction in IOP ^31^. A recent review of 498 patients undergoing phaco-trabectome surgery demonstrated a greater reduction in IOP in more severe glaucoma, as well as steroid-induced glaucoma ^32, 33^ and pseudoexfoliative glaucoma ^34, 35^. The focus on a percentage reduction is misleading because of the increase of outflow facility, which is only limited by post-trabecular outflow resistance. Patients with a high preoperative IOP can be expected to drop toward a similar postoperative IOP, near 16 mmHg, as patients with a lower preoperative IOP ^32, 36^. Non-matched studies of phaco-trabectome and trabectome outcomes do not take into account that the second group has an IOP reduction as the primary indication and a higher baseline compared to the group with cataract surgery patients, many of which may have stable glaucomas, but would like to take advantage of reduced eye drop dependency.

***AIT alone in pseudophakic or phakic eyes.*** Trabectome surgery alone can be a useful alternative to more invasive procedures in patients who have already undergone cataract surgery, as well as those who do not have a visually significant cataract. The lens status or performance of phacoemulsification in the same session has no significant impact ^37, 38^ on IOP reduction. In a review of 235 pseudophakic patients undergoing trabectome surgery compared to 352 patients undergoing phaco-trabectome, individuals with phaco-trabectome had only slightly lower IOPs (0.73 +/- 0.32 mm Hg) than patients undergoing trabectome alone ^37^.

In a prospective study of 261 patients undergoing trabectome or phaco-trabectome, there was a trend toward a greater benefit in phaco-trabectome compared to trabectome alone in phakic or pseudophakic eyes ^39^. However, in a review of 255 phakic patients undergoing trabectome compared with 498 patients undergoing phacoemulsification combined with trabectome, phakic patients had a 21% reduction in IOP compared to an 18% reduction in patients undergoing phacoemulsification. There was no statistically significant difference in IOP or the number of medications between the groups, suggesting that phacoemulsification itself may not contribute significantly to pressure lowering in these patients ^38^.

***Goniosynechialysis and AIT in narrow angles and angle closure.*** Previously, angle-based glaucoma surgery in patients with narrow angles has been thought more likely to result in synechiae and fibrosis, and this has been considered a relative contraindication to trabectome surgery. However, a retrospective review of 671 patients undergoing either trabectome or phaco-trabectome offers evidence that trabectome surgery can be successful even in these patients. Patients with an angle judged as Shaffer grade 2 or less (narrow) had a 42% reduction in IOP at one year after trabectome surgery and a 24% reduction in IOP at one year after phaco-trabectome. Similarly, patients with an angle judged as Shaffer grade 3 or above (open) had a 37% reduction in IOP at one year after trabectome surgery and a 25% reduction in IOP at one year after phaco-trabectome. There was no statistically significant difference between the groups in IOP, the number of medications, or success rates, suggesting that trabectome surgery is a viable option for patients with narrow angles ^40^.

***Failed trabeculectomy or tube shunt.*** Reoperations after failed trabeculectomy or tube shunt are some of the most challenging surgeries for a glaucoma specialist. Trabectome surgery is a suitable, minimally invasive alternative to a revision or repeat filter or shunt. In a retrospective review of 20 patients undergoing trabectome surgery after prior failed tube shunt, there was a statistically significant reduction in IOP from 23.7 +/- 6.4 mm Hg pre-op to 15.5 +/- 3.2 mm Hg at 12 months. The authors report a 84% success rate at 12 months, with only three patients requiring further surgery ^41^.

In a retrospective review of 73 patients undergoing trabectome surgery after failed trabeculectomy, there was a 28% reduction in IOP following trabectome and a 19% reduction following phaco-trabectome, with a 1-year probability of success of 81% for trabectome and 87% for phaco-trabectome ^42^. Another recent review of 60 patients undergoing trabectome surgery after failed trabeculectomy demonstrated a 36% reduction in IOP and a 14% decrease in the number of IOP-lowering medications, with 25% of patients requiring further surgery in the course of follow-up ^43^. Although these studies are limited by their retrospective nature and the relatively small number of patients included, the results suggest that the distal outflow tract is patent and functioning, contradicting the assumption that an unused outflow system atrophies. Presumably, this was the product of a misinterpretation of the high IOP that often follows when the drainage cleft of a cyclodialysis, a historical surgery now rarely performed, closes ^44– 46^. The cause of the lack of conventional outflow is instead the failure of the TM to allow fluid passage after extended periods of having been bypassed.

***Adjuvant AIT at the time of tube shunt implantation.*** Finally, with its favorable safety profile, trabectome surgery may be a valuable adjuvant at the time of both valved and non-valved tube shunt implantation. In a matched comparison of 117 patients undergoing Baerveldt tube implantation alone versus 60 patients undergoing Baerveldt combined with trabectome surgery, both groups showed similar IOPs and visual acuities at each postoperative time point. However, the combination group required fewer IOP-lowering drops at each time point than the group receiving tube alone. Therefore, adjuvant trabectome surgery may improve the quality of life of patients by reducing medication burden ^47^. Results from Ahmed tube implantations were similar ^48^; trabectome surgery plus two medications reduced IOP by 12 mmHg while Ahmed implantation plus four medications lowered IOP by 15 mmHg.

***AIT in severe glaucoma.*** When trabectome surgery outcomes are stratified by glaucoma severity, patients with more medications, a higher baseline IOP and worse visual field, experience a larger IOP reduction than subjects with less aggressive glaucoma ^32, 36^. We previously created a glaucoma index to capture the clinical treatment resistance and the risk by combining baseline IOP, the number of medications that achieved this pressure and the visual field status ^32, 36, 49^. Our experience with trabecular ablation after failed trabeculectomy suggested that removal of a highly impaired TM might have a bigger effect than the removal of an only mildly impaired TM. Analysis of 843 patients indicated that patients in the most advanced glaucoma group had a threefold larger IOP reduction than the ones in the mild glaucoma group ^36^. Due to the increasing glaucoma severity in the four glaucoma severity groups, we created, individuals with advanced glaucoma had a lower success rate of 71% compared to patients with mild glaucoma, who had a success rate of 90%. Such risk of failure needs to be taken into consideration when trabectome surgery is selected to avoid a more complication prone, traditional procedure. Otherwise, traditional filtering surgeries remain a good option to advance treatment after failed trabectome surgery ^50^, while choosing noninvasive selective laser trabeculoplasty is less successful in this situation ^51^. Age, Hispanic ethnicity, steroid-induced glaucoma and cup disk ratio were also found to be significantly associated with a greater IOP reduction ^36^. An analysis of stratified phaco-trabectome outcomes showed that worse glaucoma, pseudoexfoliation and steroid-induced glaucoma contributed to IOP reduction, but not ethnicity nor the cup disk ratio ^49^. To discover additional factors from an increased testing power using more patients, we performed a combined analysis of 1340 phaco-trabectome and trabectome patients by glaucoma severity ^32^, due to the negligible impact of phacoemulsification on IOP ^37, 38^. This study was consistent with the more limited analysis of 843 patients ^36^ and indicated a fourfold larger IOP reduction in patients of the worst glaucoma group. We found that patients of Hispanic ethnicity had an IOP reduction, which was nearly 4 mmHg greater. In addition, pseudoexfoliation and steroid glaucoma imparted an IOP reduction that was greater by 3 mmHg and 4 mmHg, respectively, than POAG ^32^. In contrast to the trabectome glaucoma severity analysis, the cup disk ratio was not significantly associated with IOP reduction in the expanded study combining trabectome with phaco-trabectome patients.

### Complications

In general, the trabectome has a highly favorable safety profile, particularly in comparison to incisional glaucoma surgery. According to a recent meta-analysis ^26^, the most common complications of trabectome surgery include hyphema (up to 100%), peripheral anterior synechiae (14%), corneal injury (6%) and temporary IOP spike (6%). Less common complications include transient hypotony, lasting less than 3 months (1.5%), iris injury (1%), cystoid macular edema (1.5% when combined with phacoemulsification), and cataract progression (1.2%). Among all 3828 patients reported by the meta-analysis, there are case reports of a few, rare complications, including cyclodialysis cleft (2 cases), aqueous misdirection (4 cases), choroidal hemorrhage (1 case), and endophthalmitis (1 case) ^26^.

## Conclusions

Trabectome surgery is a durable surgical technique that may apply to a wide range of glaucomas, including narrow-angle glaucoma and severe glaucoma after failed incisional surgery. While it has not replaced filtration or tube shunt surgery, it is a valuable addition to a previously limited surgical toolkit in the treatment of glaucoma. The combination of canalograms to estimate localized outflow function and virtual casting by reconstructing vascular spaces with SD-OCT provides new tools to investigate mechanisms and failure of outflow enhancement. New surgeons can practice angle surgery in a pig eye training model that allows measurement of progress. Learning curves from junior surgeons suggest that they are safe surgeons after 5 to 7 eyes and learn to master the technique after about 30 eyes.
