# Supplementary material for: Training strategies and outcomes of ab interno trabeculectomy with the trabectome
Source: F1000Res. 2017 May 2;6:67. Originally published 2017 Jan 23. [Version 2] doi: 10.12688/f1000research.10236.2 (PMC5428488; doi:10.12688/f1000research.10236.2)
Supplement: Peer review history

# Peer review: Referee response for version 2

**DOI:** 10.5256/f1000research.12423.r22524

**Published:** 2017-05-05

**Authors:** John T. Lind

**Affiliations:** 1 Department of Ophthalmology and Visual Sciences, Washington University School of Medicine in St. Louis, St. Louis, MO, USA

I would like to congratulate Falleno *et al.* for their fantastic work encompassing a comprehensive review of the Trabectome - utility in unique and common circumstances, using OCT to measure outflow after treatment and describing a training modality to enhance surgeon readiness to increase success and avoid complication when adapting this surgical technique. I might add that in practicing the surgical technique and using the intraoperative gonioprism, surgeons can introduce a blunt cannula to simulate introducing the handpiece in the eye to better visualize how an instrument looks under gonioscopic view.

 This article also advocates an extensive pig eye experience which can be incredibly useful. The authors site the training decreases operative time and likely potential for complications with more advanced, pre-procedure training.

 I also commend the authors for explaining the use of trabectome in non traditional indications. Trabectome has the advantage of not implanting hardware and a potential for lowing IOP with a lower risk profile than traditional glaucoma surgery. It is important to recognize that the trabectome likely only treats one area of resistance where more distal resistance in some patient might limit the benefit of this surgical technique.

I have read this submission. I believe that I have an appropriate level of expertise to confirm that it is of an acceptable scientific standard.

# Peer review: Referee response for version 1

**DOI:** 10.5256/f1000research.11025.r19579

**Published:** 2017-02-15

**Authors:** Randolf A. Widder

**Affiliations:** 1 St. Martinus-Krankenhaus Düsseldorf, Düsseldorf, Germany

This well written paper is an extensive and excellent review of the outcome of glaucoma surgery after trabectome procedure worth publishing. Also training strategies are reported like performing surgery in a pig´s eye model and practicing visualisation of the trabecular meshwork in cataract patients.

 Some minor remarks

 The authors claim, that 180 degrees of the trabecular meshwork is removed during surgery (see "Surgical and training methodologies“). This is probably not the method which is used by all trabectome surgeons. They should explain the guidelines of the manufacturing company or the experience from the early studies where the removal of less trabecular meshwork was used or advised.

 Most of the literature is based on a large databank managed by the manufacturing company. Only few patient series exist which are not part of this databank. This could be commented by the authors and they could point out or stress which papers do not rely on the Neomedix databank.

 The authors estimate that a trainee needs to perform surgery in 29 eyes to master the technique. To enable the reader to follow this interesting statement, the estimation process could be explained in more detail.

 The index of the literature should be revised between numbers 51-55. The numbers are not correct.

I have read this submission. I believe that I have an appropriate level of expertise to confirm that it is of an acceptable scientific standard.
